# Determinants of Multidrug Resistance Among Gram‐Positive Uropathogens in Central Portugal: A 5‐Year Retrospective Study

**DOI:** 10.1155/ijm/1664980

**Published:** 2026-07-19

**Authors:** Muhammad Adnan, Patrícia Coelho, Sónia Mateus, Miguel Castelo Branco, Francisco Rodrigues

**Affiliations:** ^1^ Health Research Institute, National Institute of Health, Islamabad, Pakistan, iss.it; ^2^ Sport Physical Activity and Health Research and Innovation Center (SPRINT), Polytechnic University of Castelo Branco, Castelo Branco, Portugal; ^3^ Polytechnic University of Castelo Branco, Castelo Branco, Portugal; ^4^ Faculty of Health Sciences, The University of Beira Interior, Covilha, Portugal

**Keywords:** antibacterial agents, antimicrobial stewardship, Gram-positive cocci, Portugal, urinary tract infections

## Abstract

Urinary tract infections (UTIs) are among the most common bacterial infections worldwide. Although Gram‐negative bacteria predominate, Gram‐positive cocci (GPC) are increasingly recognized as significant uropathogens. This retrospective study analyzed 655 patients with culture‐confirmed GPC UTIs in a hospital in Central Portugal (2018–2022) to identify predictors of multidrug resistance (MDR). MDR was defined as resistance to three or more antimicrobial classes. The overall MDR prevalence was 83.7%, with *Enterococcus* spp. showing the highest MDR prevalence (98.2%). Multivariate analysis identified older age as an independent predictor of MDR, whereas *Staphylococcus* and *Streptococcus* species were associated with significantly lower odds compared to *Enterococcus*. A temporal increase in MDR risk was observed in 2021 and 2022. Despite the high overall resistance rates observed, oxazolidinones and cyclic lipopeptides retained near‐complete activity against all GPC isolates, reinforcing their role as last‐line therapeutic options.

## 1. Introduction

Urinary tract infections (UTIs) are among the most common bacterial infections worldwide, representing a substantial burden for both community and hospital healthcare systems [1–3]. Although the epidemiology of UTIs has been historically dominated by Gram‐negative bacilli (GNB), there is growing recognition of the clinical relevance of Gram‐positive cocci (GPC) as significant uropathogens [4–6]. The clinical relevance of GPC is increasingly recognized in specific populations, including elderly individuals, hospitalized patients, pregnant women, and those with indwelling urinary catheters or underlying comorbidities [7–9].

Clinically important Gram‐positive uropathogens include *Enterococcus faecalis*, *Staphylococcus saprophyticus*, and *Streptococcus agalactiae*, which are frequently implicated in both uncomplicated and complicated UTIs [6, 10]. These organisms are associated with distinct virulence mechanisms, including enhanced adherence to uroepithelial cells and biofilm formation, contributing to persistent and recurrent infections [11, 12]. Consequently, current European Association of Urology (EAU) guidelines emphasize the importance of targeted antimicrobial therapy based on culture and susceptibility testing rather than empirical broad‐spectrum treatment [13].

In parallel with their increasing clinical importance, Gram‐positive uropathogens have demonstrated a concerning rise in antimicrobial resistance. Multidrug resistance (MDR), typically defined as resistance to three or more antimicrobial classes, represents a critical challenge for effective infection management [14, 15]. While resistance patterns in GNB have been extensively studied, comparatively limited attention has been given to MDR determinants among GPC, particularly in specific national contexts such as Portugal [16, 17].

Available Portuguese studies have predominantly focused on the prevalence and resistance profiles of GNB in UTIs, consistently identifying *Escherichia coli* and *Klebsiella pneumoniae* as the leading pathogens [18–21]. Nevertheless, GPC such as *Enterococcus faecalis* and *Streptococcus agalactiae* have also been reported with increasing frequency, especially in vulnerable populations, including pregnant women [20]. Moreover, recent evidence highlights a high burden of MDR among uropathogens, with extended‐spectrum *β*‐lactamase (ESBL)–producing organisms and nonfermenting GNB identified as major contributors [21–23].

Despite these insights, there remains a clear gap in the literature regarding the clinical and microbiological determinants of MDR specifically among Gram‐positive uropathogens within the Portuguese healthcare setting. To the best of our knowledge, no Portuguese studies have specifically evaluated independent predictors of MDR among Gram‐positive uropathogens using multivariable modeling approaches.

Therefore, the present study was aimed at identifying independent clinical and microbiological predictors of MDR among GPC isolated from urinary tract infections in Central Portugal (ITUCIP). We hypothesized that demographic factors, particularly age, and microbiological characteristics, especially bacterial species, are independently associated with MDR. By applying multivariable logistic regression analysis to a large retrospective dataset, this study seeks to provide a comprehensive characterization of resistance patterns and associated risk factors in this population.

## 2. Materials and Methods

### 2.1. Study Design and Setting

This retrospective observational study was conducted using data from a hospital in Central Portugal. The analysis included patient records collected over a 5‐year period, from January 2018 to December 2022, within the framework of the ITUCIP project.

### 2.2. Study Population and Sample Selection

A total of 661 patients with culture‐confirmed UTIs were included. Culture‐confirmed UTI was defined as growth of ≥ 10^5^ CFU/mL of a single GPC isolate in a properly collected urine sample, according to routine hospital microbiology laboratory criteria. Patients with isolation of GPC, including *Enterococcus*, *Staphylococcus*, and *Streptococcus* species, were eligible for inclusion.

Patients of all ages, sexes, and clinical settings were considered. Cases with incomplete antimicrobial susceptibility data (*n* = 6) were excluded, resulting in a final sample of 655 patients included in the analysis.

### 2.3. Variables and Data Collection

The dataset included demographic, clinical, and microbiological variables, namely, age, sex, pregnancy status, patient care setting, hospital department/specialty, bacterial isolate, antimicrobial susceptibility test (AST) results, and year of urine culture.

Age was categorized into five groups: < 18, 18–35, 36–55, 56–75, and > 75 years. Sex and pregnancy status were combined into a single variable with three categories: male, nonpregnant female, and pregnant female.

Patient care settings were classified as outpatient/community, emergency department, inpatient department, day treatment, laboratory‐only, and other. Hospital departments were grouped into low‐risk/community, general/internal medicine, surgery/postsurgery, high‐risk (including ICU and transplant units), urology/nephrology, and other specialties.

Bacterial isolates were categorized into six groups: *Enterococcus faecalis*, other *Enterococcus* spp., methicillin‐sensitive *Staphylococcus aureus* (MSSA), methicillin‐resistant *Staphylococcus aureus* (MRSA), coagulase‐negative staphylococci (CoNS), and *Streptococcus* spp.

### 2.4. AST

AST was performed using both disk diffusion and automated methods (VITEK 2, bioMérieux, Lyon, France). Results were interpreted according to the European Committee on Antimicrobial Susceptibility Testing (EUCAST) guidelines, Version 12.0 (2022) [24]. To ensure consistency across the study period, antimicrobial susceptibility results were reviewed and interpreted according to the EUCAST criteria adopted by the hospital laboratory.

Twelve antimicrobial classes were considered in the analysis: penicillins, beta‐lactam/beta‐lactamase inhibitors, antistaphylococcal agents, carbapenems, macrolides/lincosamides, aminoglycosides, tetracyclines, oxazolidinones, glycopeptides, cyclic lipopeptides, fluoroquinolones, and other agents (including UTI‐specific or topical agents).

### 2.5. Definition of MDR

MDR was defined as resistance to at least one antimicrobial agent in three or more antimicrobial classes. MDR classification was based on antimicrobial susceptibility results available in the laboratory database. Intrinsic resistance patterns were not separately adjusted in the MDR algorithm and were considered during interpretation of results, particularly for *Enterococcus* spp. [25]. Isolates with incomplete antimicrobial susceptibility profiles were included in the analysis. MDR classification was based on the resistance patterns observed across the antimicrobial classes tested for each isolate.

### 2.6. Statistical Analysis

Binary logistic regression was used to assess the association between independent variables and MDR status (binary outcome). Initially, univariate logistic regression models were performed for each covariate. Variables with a *p* value < 0.20 in univariate analysis were considered for inclusion in the multivariate model.

A multivariate logistic regression model was then constructed, including age, sex, patient care setting, hospital department, bacterial isolate, and year of culture as independent variables. Adjusted odds ratios (aORs) with 95% confidence intervals (CI) were calculated.

Multicollinearity was assessed using the variance inflation factor (VIF), and model fit was evaluated using the Hosmer–Lemeshow goodness‐of‐fit test. Statistical significance was defined as a *p* value ≤ 0.05. Model diagnostics, including multicollinearity assessment and goodness‐of‐fit evaluation, were performed to verify the adequacy of the final regression model. Multicollinearity was assessed using VIF, and model fit was evaluated using the Hosmer–Lemeshow goodness‐of‐fit test. These procedures were performed during statistical analysis to ensure the adequacy of the final regression model.

### 2.7. Ethical Considerations

The study was approved by the Ethics Committee and Data Protection Officer of the University of Beira Interior (reference: CE‐UBI‐Pj‐2023‐020, dated April 19, 2023). Given the retrospective nature of the study and the use of anonymized data, the requirement for informed consent was waived. All methods used in this study are standard clinical and laboratory procedures routinely applied in hospital settings.

## 3. Results

A total of 655 patients with UTIs caused by GPC were included in the final analysis. The mean age of the study population was 67.6 ± 23.2 years, ranging from 1 month to 101 years. The majority of patients were elderly (> 75 years), accounting for 47.3% of the sample, followed by individuals aged 56–75 years (29.6%). Male patients were more prevalent than females (58.9% vs. 41.1%), with pregnant women representing a small proportion (2.4%).

Most cases were identified in the inpatient setting (43.4%), followed by the emergency department (30.1%). Regarding clinical specialties, the highest proportion of patients was observed in general/internal medicine (41.1%). *Enterococcus* species were the most frequently isolated GPC, accounting for 66.1% of cases, with *Enterococcus faecalis* alone representing 56.2% of all isolates. A marked increase in GPC‐related UTIs was observed over time, with the highest proportion recorded in 2022 (42.6%) (Table 1).

**Table 1:** Demographic, clinical, and microbiological characteristics of the study population according to MDR status (minor discrepancies in counts between this table and Table 2 reflect rounding and classification based on antimicrobial susceptibility profiles).

Predictor	Category	Total (*N* = 655)		Non‐MDR (< 3) (*N* = 107)		MDR (≥ 3) (*N* = 548)	
Age (years)	< 18	39	6.0%	16	15.0%	23	4.2%
	18–35	41	6.3%	15	14.0%	26	4.7%
	36–55	71	10.8%	20	18.7%	51	9.3%
	56–75	194	29.6%	37	34.6%	157	28.6%
	> 75	310	47.3%	19	17.8%	291	53.1%

Sex	Male	386	58.9%	46	43.0%	340	62.0%
	Nonpregnant female	253	38.6%	52	48.6%	201	36.7%
	Pregnant female	16	2.4%	09	8.4%	07	1.3%

Patient care setting	Outpatient department	130	19.8%	38	35.5%	92	16.8%
	Emergency department	197	30.1%	29	27.1%	168	30.7%
	Inpatient department	284	43.4%	27	25.2%	257	46.9%
	Day treatment	17	2.6%	04	3.7%	13	2.4%
	Laboratory only	20	3.1%	07	6.5%	13	2.4%
	Others	07	1.1%	02	1.9%	05	0.9%

Department	Low risk/community	40	6.1%	08	7.5%	32	5.8%
	General/internal medicine	269	41.1%	27	25.2%	242	44.2%
	Surgery/postsurgery	47	7.2%	05	4.7%	42	7.7%
	High risk/ICU/transplant	49	7.5%	06	5.6%	43	7.8%
	Urology/nephrology	120	18.3%	18	16.8%	102	18.6%
	Other specialties	130	19.8%	43	40.2%	87	15.9%

Bacterial isolate	*Enterococcus faecalis*	368	56.2%	08	7.5%	360	65.7%
	*Enterococcus* spp. (others)	65	9.9%	0	0.0%	65	11.9%
	MSSA	31	4.7%	19	17.8%	12	2.2%
	MRSA	45	6.9%	0	0.0%	45	8.2%
	CoNS	91	13.9%	33	30.8%	58	10.6%
	*Streptococcus* spp.	55	8.4%	47	43.9%	08	1.5%

Year of culture test	2018	82	12.5%	20	18.7%	62	11.3%
	2019	71	10.8%	13	12.1%	58	10.6%
	2020	73	11.1%	12	11.2%	61	11.1%
	2021	150	22.9%	20	18.7%	130	23.7%
	2022	279	42.6%	42	39.3%	237	43.2%

Abbreviations: CoNS, coagulase‐negative *Staphylococci*; ICU, intensive care unit; MDR, multidrug resistance; MRSA, methicillin‐resistant *Staphylococcus aureus*; MSSA, methicillin‐sensitive *Staphylococcus aureus*.

**Table 2:** Distribution of drug resistance score in Gram‐positive cocci urinary tract infections (small differences in isolate counts compared to Table 1 are due to rounding and classification based on drug resistance scoring across antimicrobial classes).

Class	*N*	*Enterococci* (*N* = 433)		*Staphylococci* (*N* = 167)		*Streptococci* (*N* = 55)		Total (*N* = 655)	
Drug resistance score	0	0	0.0%	02	1.2%	07	12.7%	09	1.4%
	1	02	0.5%	18	10.8%	18	32.7%	38	5.8%
	2	06	1.4%	32	19.2%	22	40.0%	60	9.2%
	3	25	5.8%	31	18.6%	08	14.5%	64	9.8%
	4	118	27.3%	14	8.4%	0	0.0%	132	20.2%
	5	224	51.7%	37	22.2%	0	0.0%	261	39.8%
	6	54	12.5%	23	13.8%	0	0.0%	77	11.8%
	7	04	0.9%	04	2.4%	0	0.0%	08	1.2%
	8	0	0.0%	06	3.6%	0	0.0%	06	0.9%

### 3.1. Distribution of MDR

The overall prevalence of MDR among GPC isolates was 83.7% (*n* = 548). A clear age‐related trend was observed, with higher MDR rates in older age groups, particularly among patients aged > 75 years.

A higher proportion of MDR cases was observed among male patients compared to females (62.0% vs. 38.0%); however, this association was not confirmed in the multivariate analysis. MDR was also more frequent in hospital‐based settings, particularly among inpatients (46.9%) and patients presenting to the emergency department (30.7%). Similarly, higher MDR proportions were observed in patients managed within general/internal medicine departments.

From a microbiological perspective, *Enterococcus* species accounted for the majority of MDR cases (77.6%). The temporal distribution showed an increasing trend, with the highest number of MDR isolates recorded in 2022 (43.2%).

### 3.2. Resistance Profile by Bacterial Group

The distribution of resistance scores across bacterial groups revealed substantial variability in resistance patterns. Among *Enterococcus* isolates, nearly all demonstrated MDR, with 98.2% classified as MDR and more than half showing resistance to five antimicrobial classes.

In contrast, *Staphylococcus* species exhibited a lower, yet still considerable, MDR rate (68.9%). *Streptococcus* species showed the highest susceptibility profile, with only 14.5% of isolates classified as MDR and the majority demonstrating resistance to no more than two antimicrobial classes. A drug resistance score was calculated as the number of antimicrobial classes for which resistance was identified for each isolate. Higher scores indicate resistance to a greater number of antimicrobial classes.

### 3.3. Antimicrobial Susceptibility Patterns

AST demonstrated distinct resistance profiles across GPC groups. Oxazolidinones and cyclic lipopeptides exhibited near‐universal effectiveness across all isolates, with susceptibility rates approaching 100%.

Conversely, high levels of resistance were observed for several commonly used antimicrobial classes. *Enterococcus* species showed near‐complete resistance to penicillins (99.3%) and high resistance to macrolides/lincosamides (93.5%) and aminoglycosides (91.0%). *Staphylococcus* species demonstrated high resistance to carbapenems (100%), penicillins (86.9%), and fluoroquinolones (81.4%). In contrast, *Streptococcus* species maintained high susceptibility to penicillins (98.2%), although resistance to tetracyclines (74.1%) and fluoroquinolones (55.6%) remained notable (Table 3).

**Table 3:** Susceptibility and resistance patterns of Gram‐positive cocci across antimicrobial classes.

Class	*N*	Status	*Enterococci*		*Staphylococci*		*Streptococci*		Total	
Penicillins	648	Susceptible	03	0.7%	21	13.1%	54	98.2%	78	12.0%
		Resistant	430	99.3%	139	86.9%	01	1.8%	570	88.0%
Beta‐lactam/inhibitor	118	Susceptible	—	—	53	44.9%	—	—	53	44.9%
		Resistant	—	—	65	55.1%	—	—	65	55.1%
Antistaphylococcal	143	Susceptible	—	—	72	50.3%	—	—	72	50.3%
		Resistant	—	—	71	49.7%	—	—	71	49.7%
Carbapenems	06	Susceptible	—	—	0	0.0%	—	—	0	0.0%
		Resistant	—	—	06	100.0%	—	—	6	100.0%
Macrolides/lincosamides	651	Susceptible	28	6.5%	66	39.5%	36	70.6%	130	20.0%
		Resistant	405	93.5%	101	60.5%	15	29.4%	521	80.0%
Aminoglycosides	491	Susceptible	29	9.0%	131	78.4%	—	—	160	32.6%
		Resistant	295	91.0%	36	21.6%	—	—	331	67.4%
Tetracyclines	654	Susceptible	76	17.6%	148	88.6%	14	25.9%	238	36.4%
		Resistant	357	82.4%	19	11.4%	40	74.1%	416	63.6%
Oxazolidinones	654	Susceptible	433	100.0%	166	99.4%	54	100.0%	653	99.8%
		Resistant	0	0.0%	01	0.6%	0	0.0%	01	0.2%
Glycopeptides	654	Susceptible	420	97.0%	161	96.4%	54	100.0%	635	97.1%
		Resistant	13	3.0%	06	3.6%	0	0.0%	19	2.9%
Cyclic lipopeptides	648	Susceptible	428	100.0%	163	98.2%	54	100.0%	645	99.5%
		Resistant	0	0.0%	03	1.8%	0	0.0%	3	0.5%
Fluoroquinolones	654	Susceptible	279	64.4%	31	18.6%	24	44.4%	334	51.1%
		Resistant	154	35.6%	136	81.4%	30	55.6%	320	48.9%
Others/UTI/topical	631	Susceptible	53	12.3%	120	71.9%	32	100.0%	205	32.5%
		Resistant	379	87.7%	47	28.1%	0	0.0%	426	67.5%

### 3.4. Univariate Analysis of MDR Predictors

Univariate logistic regression analysis identified several factors associated with MDR. Increasing age was strongly associated with higher MDR risk, with patients aged 56–75 years (OR = 2.952; *p* = 0.004) and > 75 years (OR = 10.654; *p* < 0.001) showing significantly elevated odds.

Female sex was associated with a reduced likelihood of MDR, both among nonpregnant women (OR = 0.523; *p* = 0.003) and pregnant women (OR = 0.105; *p* < 0.001). The clinical setting also influenced MDR risk, with significantly higher odds observed in the inpatient (OR = 3.932; *p* < 0.001) and emergency department settings (OR = 2.393; *p* = 0.002).

Microbiologically, *Staphylococcus* (OR = 0.042; *p* < 0.001) and *Strep*tococcus (OR = 0.003; *p* < 0.001) were associated with significantly lower odds of MDR compared to *Enterococcus*. Temporal analysis indicated an increasing trend in MDR risk, particularly in 2021 (OR = 2.097; *p* = 0.035) and borderline significance in 2022 (Table 4).

**Table 4:** Univariate regression analysis of predictors associated with multidrug‐resistant Gram‐positive cocci.

Predictor	Category	Exp(B)	95% CI for Exp(B)		Sig.
			Lower	Upper	
Age (years)	< 18	REF			
	18–35	1.206	0.490	2.967	0.684
	36–55	1.774	0.780	4.033	0.171
	56–75	2.952	1.420	6.136	0.004
	> 75	10.654	4.840	23.452	< 0.001

Sex	Male	REF			
	Nonpregnant female	0.523	0.339	0.807	0.003
	Pregnant female	0.105	0.037	0.296	< 0.001

Patient care setting	Outpatient department	REF			
	Emergency department	2.393	1.386	4.131	0.002
	Inpatient department	3.932	2.273	6.799	< 0.001
	Day treatment	1.342	0.411	4.380	0.626
	Laboratory only	0.767	0.284	2.072	0.601
	Others	1.033	0.192	5.556	0.970

Department	Low risk/community	REF			
	General/internal medicine	2.241	0.938	5.353	0.069
	Surgery/postsurgery	2.100	0.627	7.030	0.229
	High risk/ICU/transplant	1.792	0.566	5.676	0.322
	Urology/nephrology	1.417	0.563	3.564	0.459
	Other specialties	0.506	0.215	1.191	0.119

Bacterial isolate	*Enterococci*	REF			
	*Staphylococci*	0.042	0.019	0.090	< 0.001
	*Streptococci*	0.003	0.001	0.009	< 0.001

Year of culture test	2018	REF			
	2019	1.439	0.657	3.154	0.363
	2020	1.640	0.738	3.643	0.225
	2021	2.097	1.052	4.179	0.035
	2022	1.820	0.998	3.321	0.051

### 3.5. Multivariate Analysis of MDR Predictors

In the multivariate model, age remained an independent predictor of MDR. Patients aged > 75 years had a significantly increased risk (aOR = 9.532; *p* = 0.004), whereas individuals aged 36–55 years also showed elevated odds (aOR = 4.729; *p* = 0.048). Among all variables included in the model, bacterial genus emerged as the strongest independent predictor of MDR, with markedly lower odds observed among *Staphylococcus* and *Streptococcus* isolates compared with *Enterococcus*.

The associations previously observed for sex and clinical setting were no longer statistically significant after adjustment. In contrast, the bacterial isolate remained a strong independent predictor, with *Staphylococcus* (aOR = 0.042; *p* < 0.001) and *Streptococcus* (aOR = 0.002; *p* < 0.001) showing markedly reduced odds of MDR compared to *Enterococcus*.

Temporal trends persisted in the adjusted analysis, with significantly higher MDR risk observed in 2021 (aOR = 3.761; *p* = 0.017) and 2022 (aOR = 2.645; *p* = 0.040) (Table 5).

**Table 5:** Multivariate regression analysis of predictors associated with multidrug‐resistant Gram‐positive cocci.

Predictor	Category	Exp(B)	95% CI for Exp(B)		Sig.
			Lower	Upper	
Age (years)	< 18	REF			
	18–35	3.452	0.685	17.392	0.133
	36–55	4.729	1.012	22.106	0.048
	56–75	2.827	0.665	12.017	0.159
	> 75	9.532	2.076	43.759	0.004

Sex	Male	REF			
	Nonpregnant female	1.336	0.618	2.888	0.461
	Pregnant female	0.162	0.019	1.385	0.096

Patient care setting	Outpatient department	REF			
	Emergency department	0.975	0.275	3.456	0.969
	Inpatient department	1.127	0.356	3.570	0.838
	Day treatment	0.234	0.037	1.485	0.123
	Laboratory only	0.672	0.083	5.406	0.708
	Others	0.325	0.024	4.402	0.398

Department	Low risk/community	REF			
	General/internal medicine	1.557	0.310	7.824	0.591
	Surgery/postsurgery	0.943	0.143	6.205	0.952
	High risk/ICU/transplant	0.616	0.101	3.738	0.598
	Urology/nephrology	1.114	0.274	4.533	0.880
	Other specialties	1.805	0.401	8.125	0.442

Bacterial isolate	*Enterococci*	REF			
	*Staphylococci*	0.042	0.018	0.099	< 0.001
	*Streptococci*	0.002	0.001	0.007	< 0.001

Year of culture test	2018	REF			
	2019	0.901	0.281	2.891	0.861
	2020	3.102	0.951	10.120	0.061
	2021	3.761	1.268	11.159	0.017
	2022	2.645	1.046	6.686	0.040

*Note:* Variable(s) entered on Step 1: age (years), sex, patient care setting, department, bacterial isolate, and year of culture test.

## 4. Discussion

The present study demonstrates a high burden of MDR among GPC‐causing UTIs, with *Enterococcus* species emerging as the predominant and most resistant pathogens. The overall MDR prevalence observed (83.7%) is higher than that reported in several comparable studies, where rates typically range between 30% and 60%, suggesting a particularly concerning resistance landscape in this population [21–23, 26].

These findings highlight the growing clinical relevance of GPC in UTIs, especially in elderly and hospital‐associated populations, where selective antibiotic pressure and repeated healthcare exposure may contribute to the amplification of resistant strains [27–30].

Notably, the present cohort is largely composed of elderly and hospitalized patients, both of which are well‐established risk factors for antimicrobial resistance. Additionally, the inclusion of a high proportion of *Enterococcus* species—organisms intrinsically associated with resistance—likely contributes to the elevated MDR burden observed [16, 31–33].

Consistent with previous studies, *Enterococcus* species were the predominant Gram‐positive uropathogens and exhibited the highest levels of resistance [33, 34]. In the present analysis, 98.2% of *Enterococcus* isolates were classified as MDR, with a substantial proportion demonstrating resistance to multiple antibiotic classes. These findings are aligned with international data highlighting the adaptive capacity of enterococci, including intrinsic resistance mechanisms and the ability to acquire resistance determinants, particularly against aminoglycosides and *β*‐lactams [35–38]. The near‐universal resistance to penicillins and the high resistance to aminoglycosides observed in this study further reinforce this concern [39–41]. The high proportion of *Enterococcus* spp. in the present cohort, together with their well‐documented intrinsic resistance to several antimicrobial classes, should be considered when interpreting MDR prevalence. Intrinsic resistance was not separately adjusted in the MDR classification algorithm and may therefore contribute to higher observed MDR rates in this genus.

In contrast, *Staphylococcus* and *Streptococcus* species demonstrated comparatively lower MDR rates, with *Streptococcus* isolates showing the highest susceptibility profiles. These results are consistent with prior Portuguese data indicating low resistance levels in *Streptococcus agalactiae*, particularly to first‐line agents such as nitrofurantoin [42, 43]. The observed differences between bacterial groups highlight the importance of species‐level identification in guiding antimicrobial therapy.

Importantly, the study identified age as a strong independent predictor of MDR, with elderly patients exhibiting a significantly increased risk. This finding is clinically plausible and supported by previous research, as older individuals are more likely to have repeated healthcare exposure, multiple comorbidities, and prior antibiotic use, all of which contribute to the selection of resistant organisms [44–46]. Interestingly, while univariate analysis suggested a protective effect of female sex, this association did not persist in the adjusted model, indicating that confounding factors may explain the initial observation. Another relevant finding is the temporal increase in MDR risk, particularly in 2021 and 2022. This trend may reflect evolving antimicrobial resistance dynamics, potentially influenced by changes in antibiotic prescribing patterns, healthcare utilization, or broader systemic factors [47–53]. Similar temporal increases in resistant Gram‐positive pathogens, including MRSA, have been documented in other European settings, suggesting that the observed pattern may be part of a wider epidemiological shift [54, 55].

From a therapeutic perspective, the study provides valuable insights into antimicrobial effectiveness. Oxazolidinones and cyclic lipopeptides demonstrated near‐complete activity against all GPC isolates, confirming their role as critical last‐line agents. These findings are consistent with previous studies reporting sustained efficacy of linezolid, daptomycin, and glycopeptides against resistant Gram‐positive pathogens [56–59]. However, the preserved activity of these agents must be interpreted cautiously, as increased reliance on last‐line therapies may accelerate the emergence of resistance if not carefully managed.

The clinical implications of these findings are substantial. The high burden of MDR, particularly among *Enterococcus* species and elderly patients, underscores the need for strengthened resistance surveillance and optimization of empirical antimicrobial therapy [60, 61]. Empirical treatment strategies should be carefully reconsidered, with greater emphasis on local resistance patterns and patient‐specific risk factors. Moreover, the results support the implementation of routine surveillance systems to monitor resistance trends and guide evidence‐based prescribing practices [61–63]. From a clinical perspective, these findings suggest that empirical treatment strategies for suspected Gram‐positive UTIs, particularly in elderly and hospitalized patients, should be carefully reconsidered. Antibiotics with high resistance rates, such as penicillins and fluoroquinolones, may have limited effectiveness in this study population, particularly given the predominance of *Enterococcus* spp [64–66].

Despite its strengths, this study has several limitations that should be acknowledged. As a retrospective, single‐center study, the findings may be influenced by local epidemiological patterns and may not be fully generalizable to other regions. Additionally, the use of secondary laboratory data limits the availability of detailed clinical information, such as prior antibiotic exposure, comorbidities, or duration of hospitalization, which are known contributors to antimicrobial resistance. Nevertheless, the study provides a robust and comprehensive analysis of MDR among GPC in a Portuguese healthcare setting, addressing an important gap in the literature. Additionally, important clinical variables such as prior antibiotic exposure, presence of urinary catheters, comorbidities, and duration of hospitalization were not available in the dataset and may act as potential confounders. Their absence may have influenced the observed associations and should be addressed in future studies. Furthermore, the predominance of *Enterococcus* spp. among isolates may limit the generalizability of the findings to other populations of Gram‐positive uropathogens.

## 5. Conclusions

This study highlights a high burden of MDR among GPC‐causing ITUCIP, with *Enterococcus* species representing the most resistant pathogens identified in this cohort. Increasing age and temporal trends were identified as independent predictors of MDR.

Despite the high overall resistance rates observed, oxazolidinones and cyclic lipopeptides retained near‐complete activity against all GPC isolates, reinforcing their role as last‐line therapeutic options. However, their preservation requires careful antimicrobial stewardship.

These findings have important clinical implications and should inform local empirical treatment guidelines, antimicrobial stewardship strategies, and resistance surveillance programs, particularly in high‐risk populations such as elderly and hospitalized patients.

## Author Contributions

Conceptualization: M.A., P.C., and F.R. Methodology: P.C. and S.M. Validation: P.C. and S.M. Formal analysis and data curation: P.C. Investigation: M.C.B. and S.M. Writing—original draft preparation: M.A. and M.C.B. Writing—review and editing: F.R. and S.M. Visualization: F.R. Supervision: F.R. Project administration: F.R.

## Funding

This study was funded by the SPRINT: Sport Physical Activity and Health Research and Innovation Center (Centro de Investigação e Inovação em Desporto Atividade Física e Saúde, Portugal) and the FCT: Fundação para a Ciência e a Tecnologia (Portuguese Foundation for Science and Technology) (UID/06185/2025 [10.54499/UID/06185/2025], UID/PRR/06185/2025 [10.54499/UID/PRR/06185/2025], and UID/PRR2/06185/2025).

## Disclosure

All authors have read and agreed to the published version of the manuscript. The statements, opinions, and data contained in all publications are solely those of the individual author(s) and contributor(s) and not of MDPI and/or the editor(s). MDPI and/or the editor(s) disclaim responsibility for any injury to people or property resulting from any ideas, methods, instructions, or products referred to in the content.

## Ethics Statement

This study was conducted in accordance with the Declaration of Helsinki and approved by the Ethics Committee of the University of Beira Interior (CE‐UBI‐Pj‐2023‐020, April 18, 2023) for studies involving humans.

## Consent

Patient consent was waived due to it being a retrospective study, with data consultation at a hospital.

## Conflicts of Interest

The authors declare no conflicts of interest.

## Data Availability

The data that support the findings of this study are available from the corresponding author upon reasonable request.
